# Supplementary figures and images for: A scalable Drosophila assay for clinical interpretation of human PTEN variants in suppression of PI3K/AKT induced cellular proliferation
Source: PLoS Genet. 2021 Sep 7;17(9):e1009774. doi: 10.1371/journal.pgen.1009774 (PMC8448351; doi:10.1371/journal.pgen.1009774)

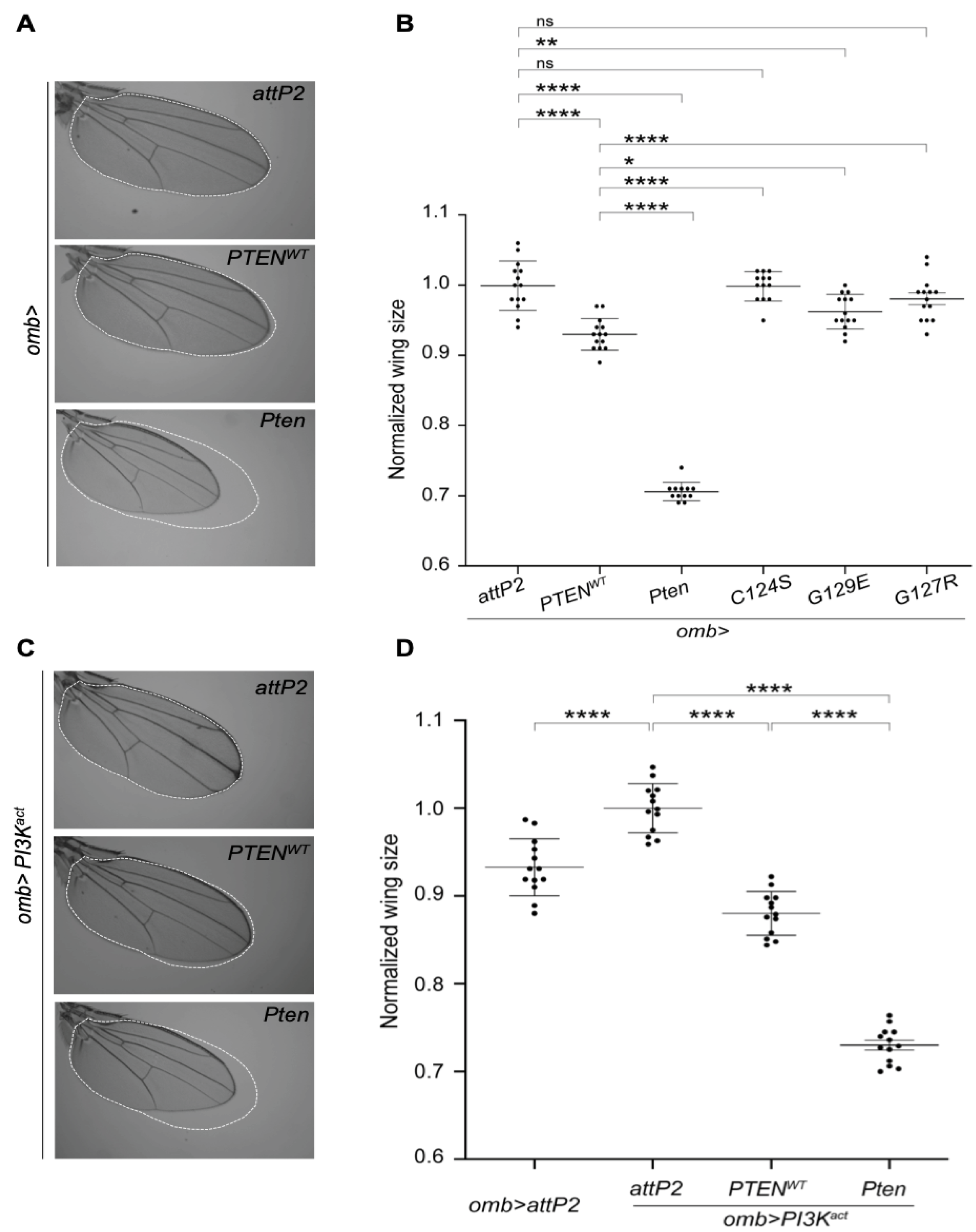

Supplement: S1 Fig — (A) Representative adult wings from flies of genotype (from top to bottom), omb>attP2, omb>PTEN and omb>dPten. (B) Normalized adult wing size data in a non-PI3K activated background for flies expressing omb>attP2, omb>PTEN, omb>dPten, omb>C124S, omb> G129E and omb> G127R. Adult wing sizes normalized by dividing individual wing area data point by the average of omb>attP2 wing area. (C) Representative adult wings from flies expressing (from top to bottom) omb>PI3Kact+attP2, omb>PI3Kact+PTEN-WT, omb>PI3Kact+dPten. (D) Normalized adult wing size data in a PI3K activated background for flies expressing omb>attP2 (no PI3K), omb>PI3Kact+attP2, omb>PI3Kact+PTEN, omb>PI3Kact+dPten. Adult wing sizes were normalized by dividing individual wing area data points by the average of omb>PI3Kact+attP2 area. Data are expressed as mean ± SD and analyzed using one-way ANOVA with post-hoc Tukey HSD; not significant (ns), * p < 0.05, ** p < 0.01, **** p < 0.0001. (TIF) [file pgen.1009774.s001.tif]

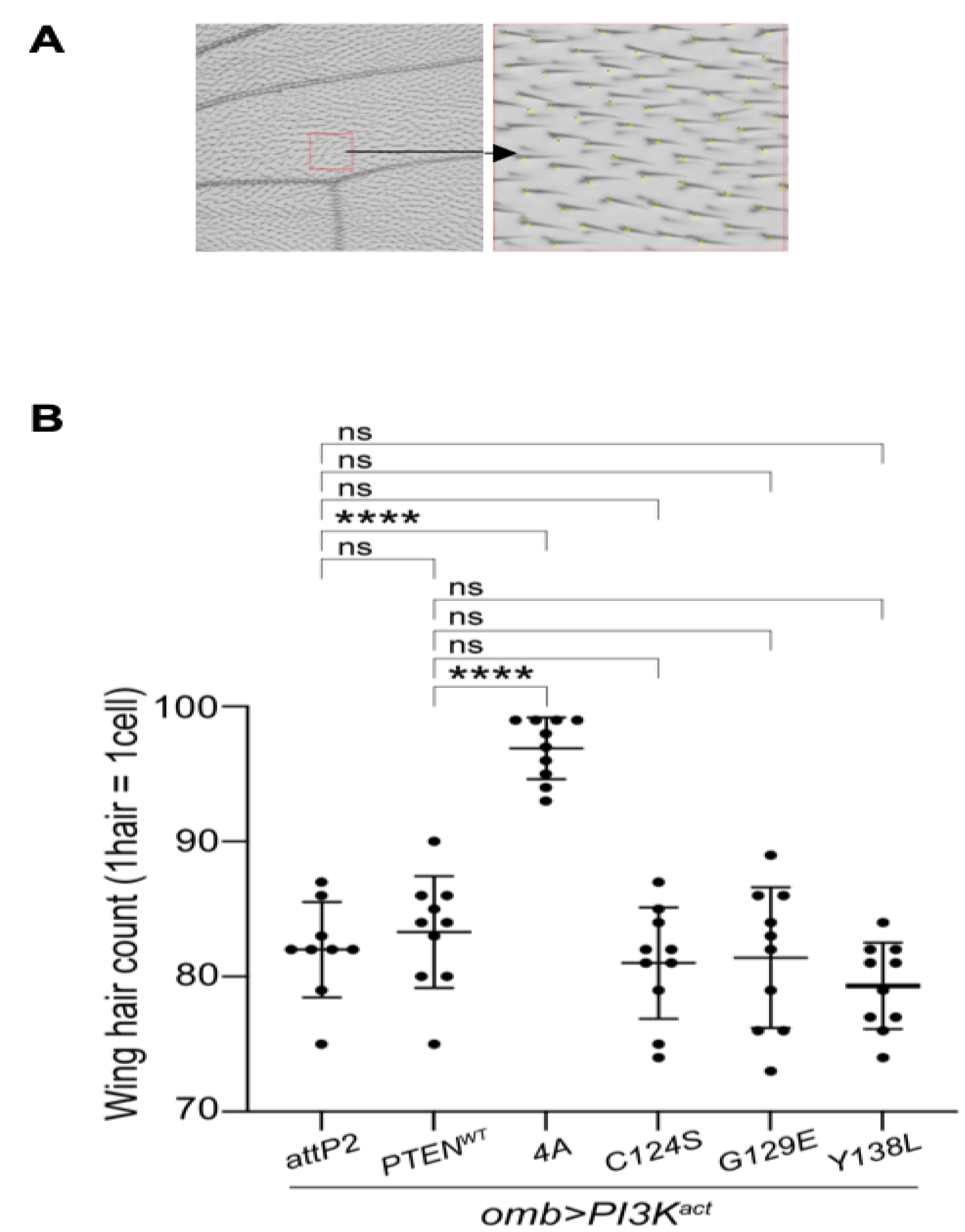

Supplement: S2 Fig — (A) Representative image of an adult wing taken at 20X magnification (left panel) and zoomed in image to show the area in which wing hairs were counted, within the L3/L4 intervein region above the posterior cross vein. (B) Graph showing quantification of wings hairs for each genotype as shown. Each datum point in scatter plots represents a single wing. Data are expressed as mean ± SD and analyzed using one-way ANOVA with post-hoc Tukey HSD; * p < 0.05, ** p < 0.01, **** p < 0.0001. ns = not significant. (TIF) [file pgen.1009774.s002.tif]

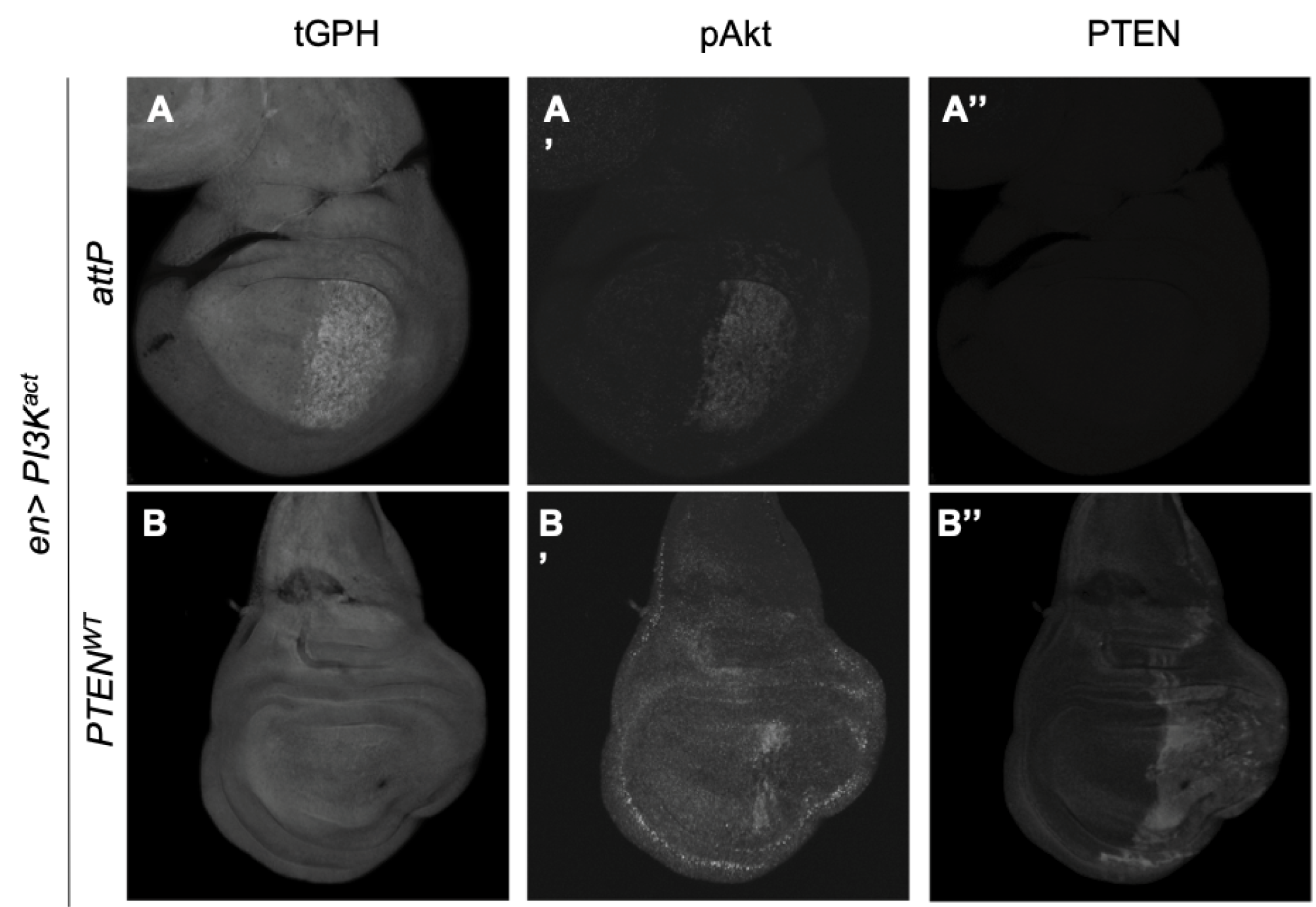

Supplement: S3 Fig — Representative images of imaginal wing discs of 3rd instar larvae stained with anti-GFP to visualize tGPH, a fluorescent sensor of PIP3 levels [74] (A,B) anti-pAkt (A’,B’) and anti-PTEN (A”,B”) for attp2 and PTEN-WT. Expression of PTEN-WT leads to suppression of both PI3K-induced PIP3 and pAkt levels in the posterior compartment of the wing imaginal disc. (TIF) [file pgen.1009774.s003.tif]

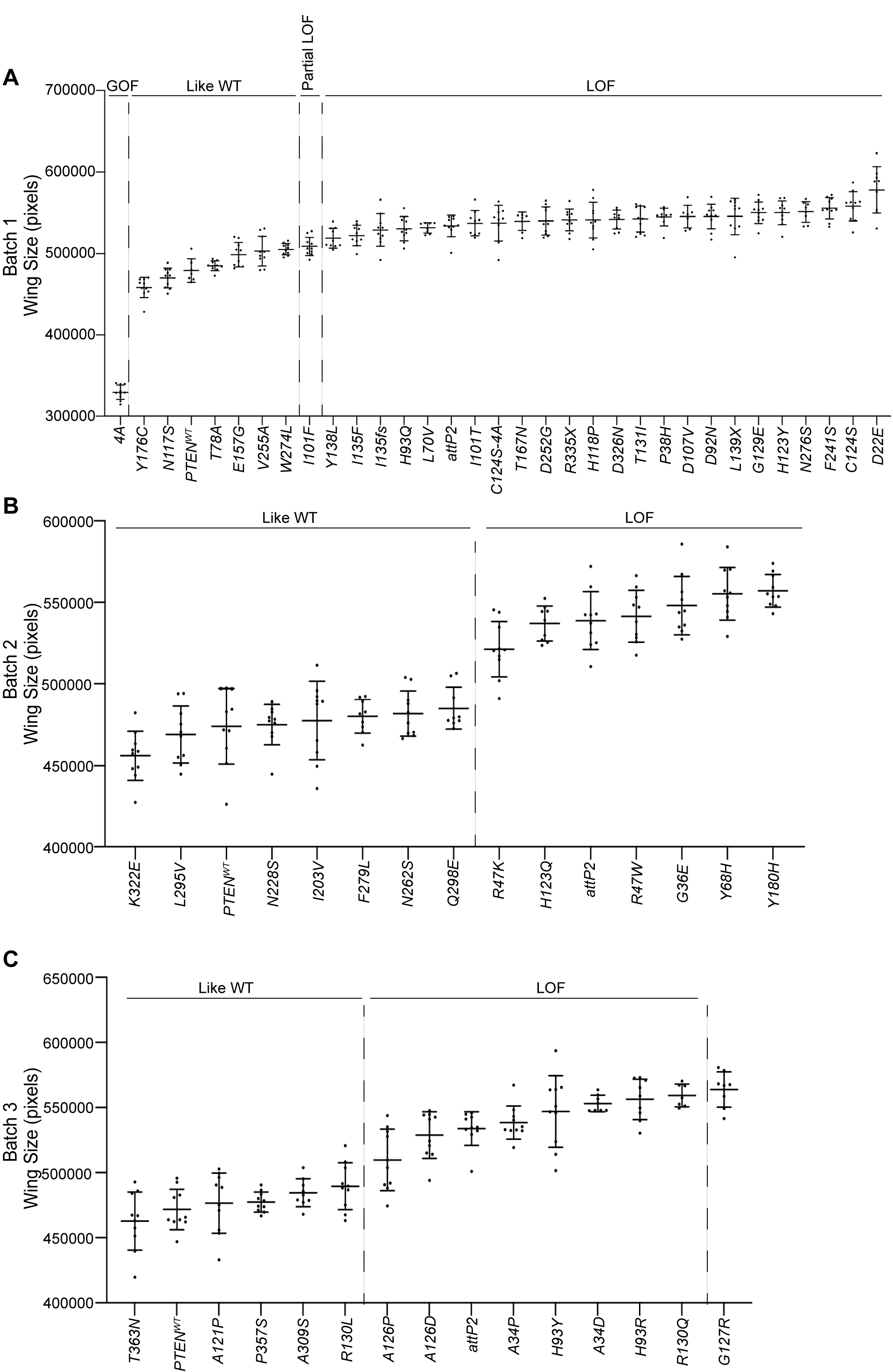

Supplement: S4 Fig — PTEN variants were tested across 7 batches total; attp2 and PTEN-WT were repeated in each batch. (A-C) Adult wing size (in pixels) of PTEN variants assayed within each batch. Variants indicated as “GOF” had significantly smaller wings than PTEN-WT, “Like WT” were not significantly different from PTEN-WT, “Partial LOF” were significantly different from both PTEN-WT and attP2. “LOF” were significantly different from PTEN-WT and not significantly different from attP2. The wing size assay demonstrates the utility of Drosophila as a model system to test the relative function of PTEN variants. Each datum point in the scatter plot represents a single adult wing. Data are expressed as mean ± SD. Significant differences as stated were obtained from analysis within each batch using one-way ANOVA with post-hoc Tukey HSD. (TIF) [file pgen.1009774.s004.tif]

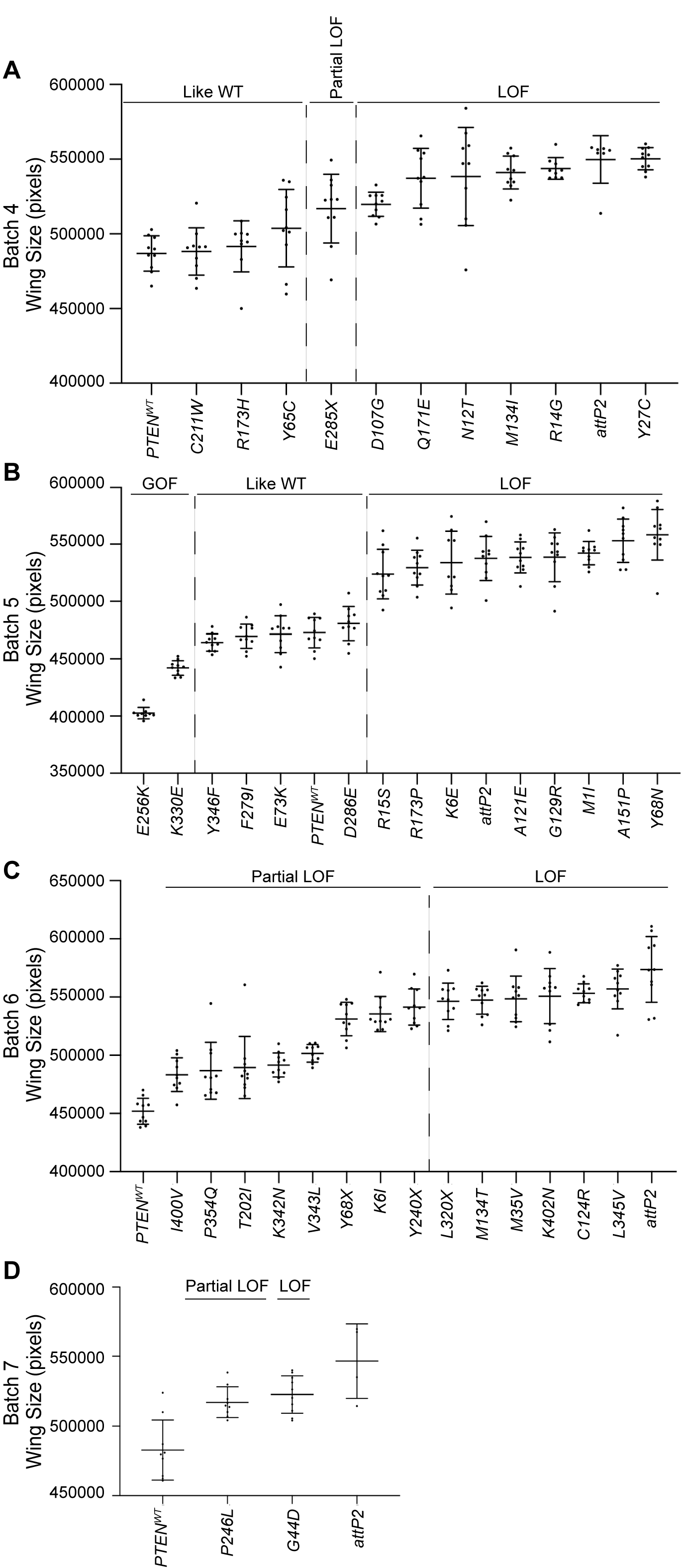

Supplement: S5 Fig — PTEN variants were tested across 7 batches total; attp2 and PTEN-WT were repeated in each batch. (A-C) Adult wing size (in pixels) of PTEN variants assayed within each batch. Variants indicated as “GOF” had significantly smaller wings than PTEN-WT, “Like WT” were not significantly different from PTEN-WT, “Partial LOF” were significantly different from both PTEN-WT and attP2. “LOF” were significantly different from PTEN-WT and not significantly different from attP2. The wing size assay demonstrates the utility of Drosophila as a model system to test the relative function of PTEN variants. Each datum point in the scatter plot represents a single adult wing. Data are expressed as mean ± SD. Significant differences as stated were obtained from analysis within each batch using one-way ANOVA with post-hoc Tukey HSD. (TIF) [file pgen.1009774.s005.tif]

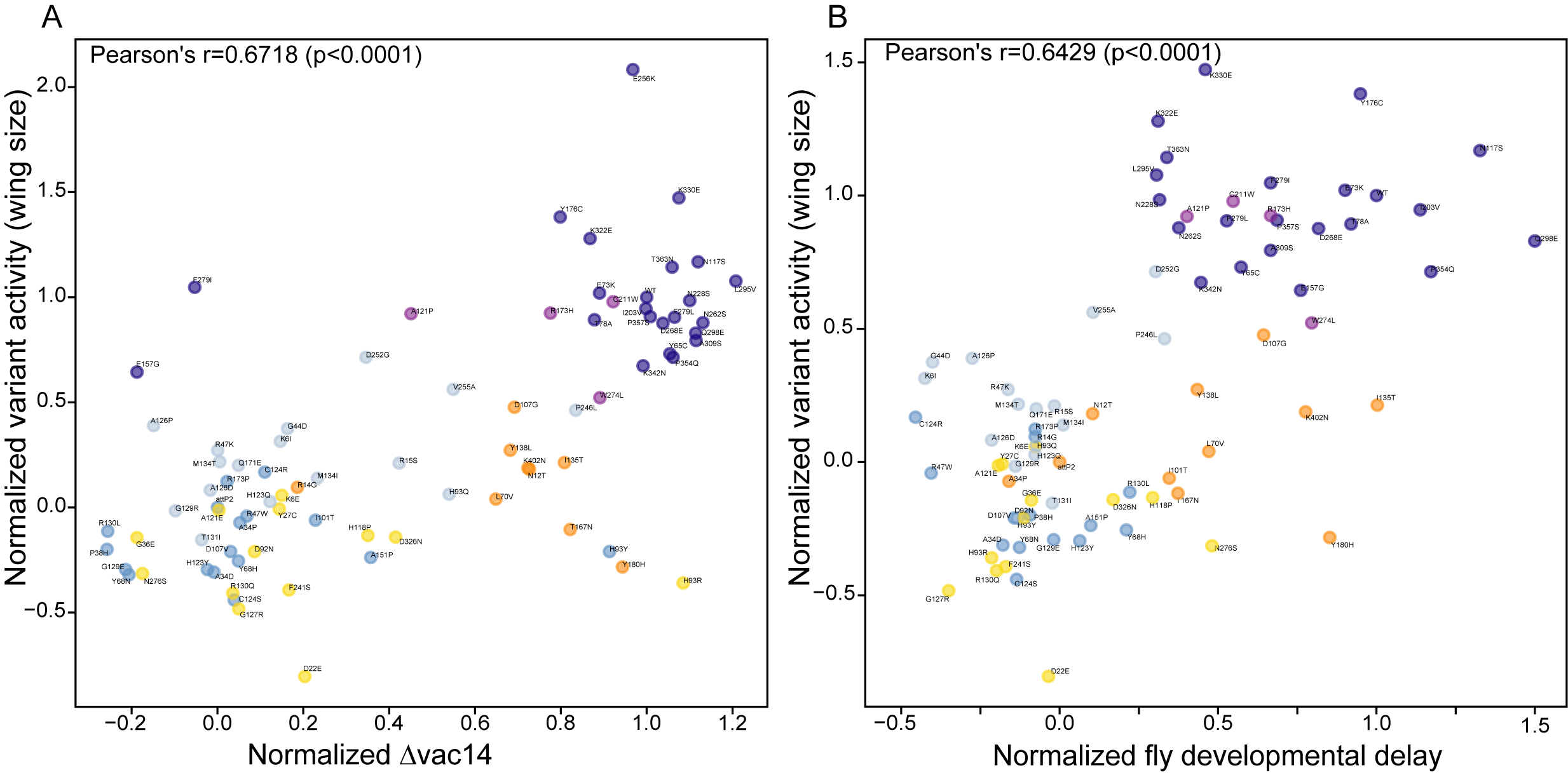

Supplement: S6 Fig — Pairwise correlation scatter plots between the wing size assay and the yeast sentinel interaction mapping of PTEN variants using the ΔVAC14 sentinel [50] (A), or the Drosophila developmental delay assay (time to eclosion) (B) [50]. Each datum point indicates an individual normalized variant (where attP2 = 0 and PTEN-WT = 1) with clusters coloured according to clusters identified in Fig 6A. (TIF) [file pgen.1009774.s006.tif]

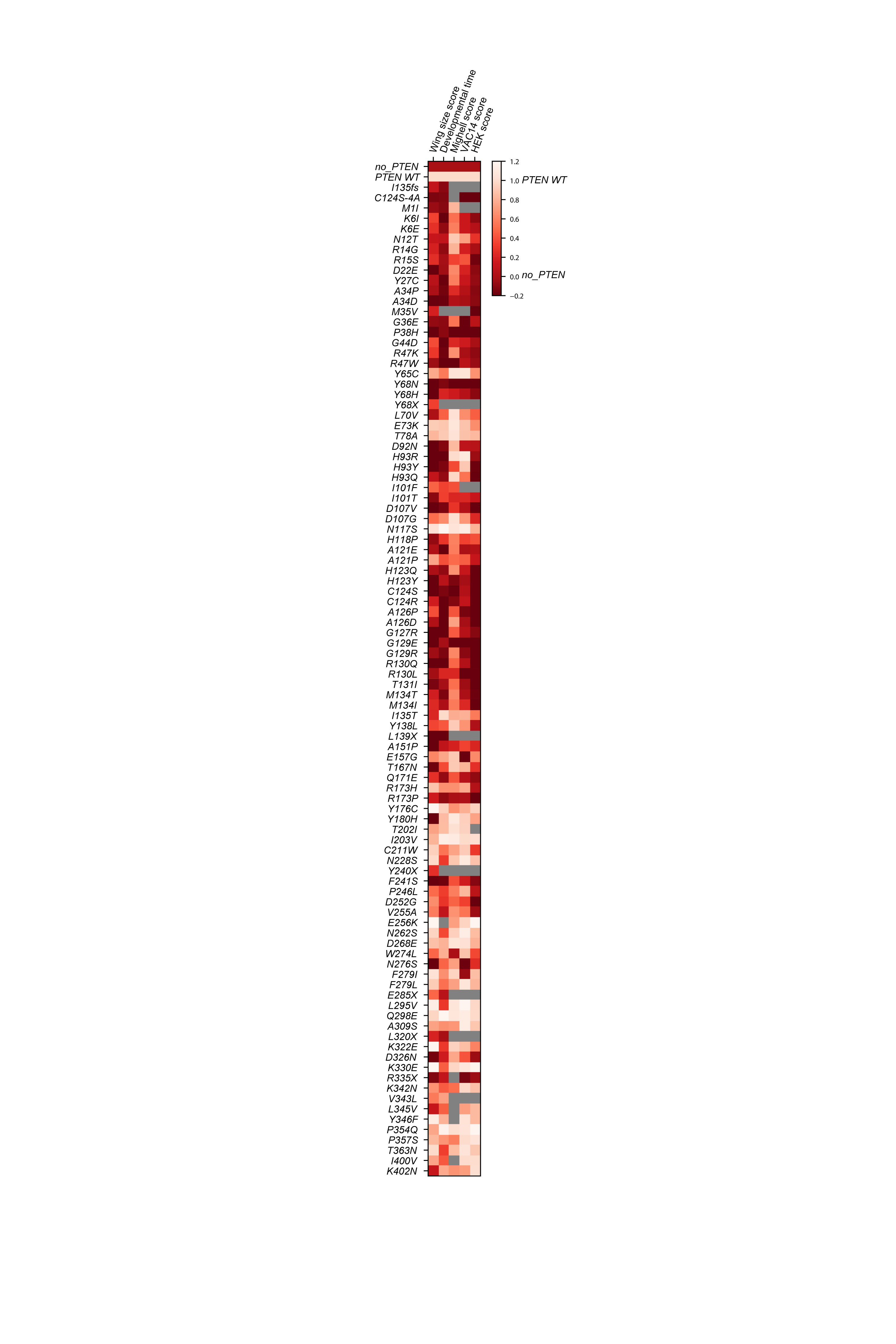

Supplement: S7 Fig — A heatmap comparing the relative function of PTEN variants in available assays; wing size assay in Drosophila, developmental delay assay (time to eclosion) in Drosophila, yeast growth dependent on PIP3 to PlP2 hydrolysis, ΔVAC14 yeast sentinel and the ratio of pAKT/AKT immunoreactivity levels in HEK (where no PTEN = 0 and PTEN-WT = 1). (TIF) [file pgen.1009774.s007.tif]

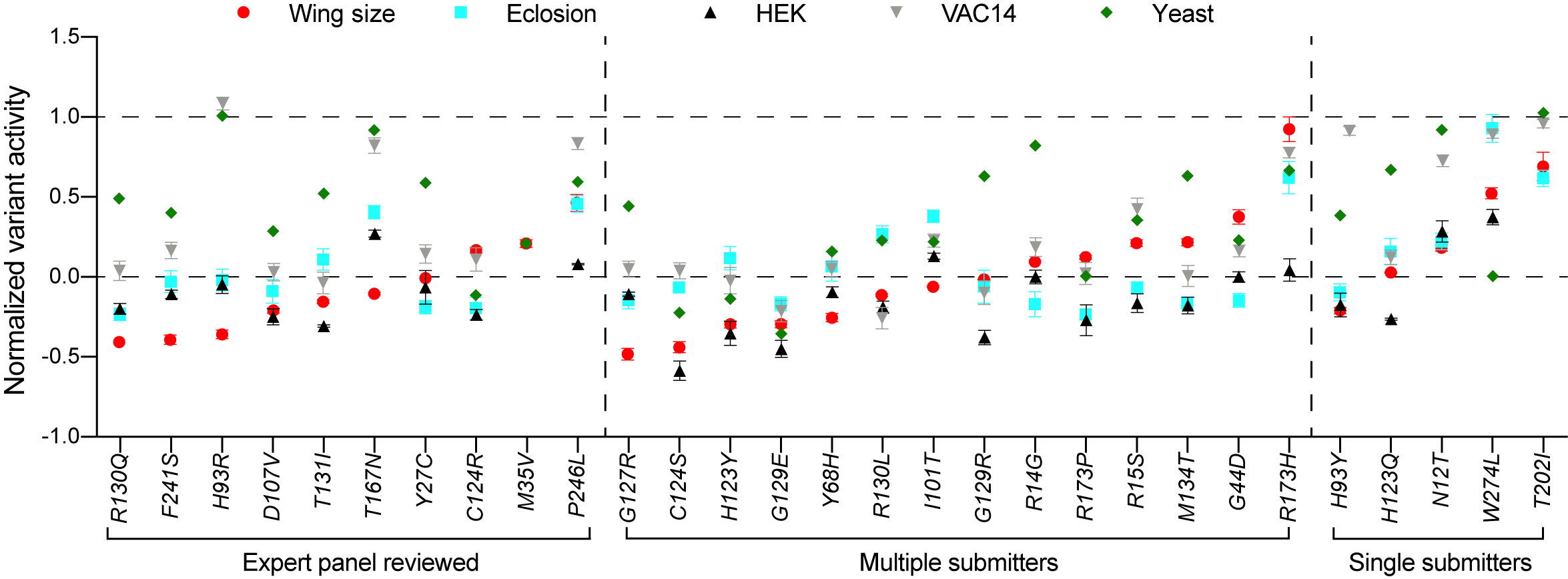

Supplement: S8 Fig — Graph showing normalized variant activity of ClinVar pathogenic PTEN variants where attP2 (no PTEN) = 0 and PTEN-WT = 1, across 5 different assays. The pathogenic variants were grouped into three categories in ClinVar; expert panel curated, multiple submitters and single submitter. Each colour indicates a particular assay. Red circles indicate the wing size assay, blue squares indicate the eclosion assay, the black triangles indicate the HEK cell assay, the grey upside-down triangles indicate ΔVAC14 yeast sentinel interaction of PTEN and the green diamonds indicate the yeast growth assay dependent on PIP3 to PlP2 hydrolysis. These data show that the wing assay performs very well across the 10 expert panel curated and 14 multiple submitters pathogenic variants. Each datum point indicates a normalized variant mean. Data are expressed as the normalized mean. (TIF) [file pgen.1009774.s008.tif]
