# Supplementary material for: A scalable Drosophila assay for clinical interpretation of human PTEN variants in suppression of PI3K/AKT induced cellular proliferation
Source: PLoS Genet. 2021 Sep 7;17(9):e1009774. doi: 10.1371/journal.pgen.1009774 (PMC8448351; doi:10.1371/journal.pgen.1009774)
Supplement: Attachment — Submitted filename: Response to reviewers.docx [file pgen.1009774.s012.docx]

**Department of Cellular and Physiological Sciences**

Faculty of Medicine

2350 Health Sciences Mall

University of British Columbia

Vancouver, B.C. V6T 1Z3

Phone 604 822 6567

Fax 604 822 2316

www.cellphys.ubc.ca	Fax 604 822 2316

www.cellphys.ubc.ca

July 15, 2021

Dear Editors and Reviewers,

We thank the reviewers for their overall support of the manuscript.

Reviewer 1. *“The authors persuasively argue for the need to implement different assays to study variant function in different cell contexts, model organisms, cell cultures, and three-dimensional organoids. I fully agree and I think the study is convincing and well done. “*

Reviewer 2. *“Overall, I feel the work to be of interest to the readership of PLoS Genetics, especially for clinical geneticists who are interested in VUS interpretation as well as Drosophila geneticists who study human diseases and genetic variants. The work is well designed and carried out with rigor. The manuscript is well-written and I feel there was a lot of work went into this study. Although the paper focuses on PTEN variants, a similar methodology can be used to study many variants in. diverse human disease relevant genes.”*

Reviewer 3 *“Overall, I think the authors have done a good job in characterising the assay and demonstrating that it has value in assessing the activity of novel variants. I am not a clinician, so I am not in a position to confirm how useful this will be, but my impression is that it will provide a robust assay that will complement other types of test in human and yeast cells.”*

Reviewer 4 *“Generally, the experiments are well controlled and described, and the authors demonstrate how this system addresses the criteria as outlined by the working groups and panels that have highlighted the need for functional assays”*

Below, we provide our response to reviewer comments (bullet points under reviewer comments), and also submit a revised version of the paper, which we hope is now more suitable for publication in PLoS Genetics.

**Comments shared by multiple reviewers**

Reviewer 1: *“My second comment/criticism relates to the statement of “tests the disease-relevant physiological context and biochemical activity of PTEN”. Despite this claim, what the authors do are tests of overexpression and hence supraphysiologic expression driven by the Gal4/UAS system and in the presence of two copies of endogenous PTEN, which is highly functionally conserved and which in my opinion combined with overexpression can be confounding factors for evaluation of certain type of mutations.”*

Reviewer 4: *“The GAL4-UAS-mediated overexpression of the normal and variant PTEN alleles is also potentially problematic. Protein levels are not examined here, and this system typically drives expression at multiples of the endogenous gene.” “the reliance on GAL4-mediated over-expression, both of PTEN and of PI3K, may undermine the in vivo relevance of these results.”*

- We believe the overarching criticism regards our poor choice of language “*relevant physiological context and biochemical activity of PTEN*”. We agree that this is problematic and have changed this language to ‘defined gene-disease mechanism’ throughout the manuscript, which is in accordance with Brnich et al (2019), and we believe is better aligned to our work. No scalable assay of variant function, which is a key goal of this report, would be able to assess variant function in a perfectly physiological context; variants would always be outside the individual’s genetic and epigenetic background. In scalable assays, the best one can achieve is to test variant function in a defined gene-disease mechanism, and to exhibit a high positive predictive rate for pathogenic variants and a high negative predictive rate for benign variants. This can be demonstrated by showing good performance for benchmarking and calibrating variants. We have established these criteria for our reported assay, notably for the first time in *Drosophila*. We have added considerable discussion of these points throughout the manuscript.
- We would also add that our study is more focused on using genetic interaction suppression assays to exclusively test human PTEN’s ability to suppress PI3K signaling levels, rather than examining the overall impact of human PTEN in *Drosophila* tissues. Such suppression assays are a powerful and selective method for isolating the activity of proteins that act within a specific pathway.
- We are not aware of any experimental functional assay, including those used by the ClinGen PTEN expert panel, that did not use non-physiological contexts and supraphysiological levels of PTEN. They either used non-physiological cell culture conditions, and/or transfection, extrachromosomal arrays or transgenics in order to overexpress PTEN variants. Gene replacement strategies could conceivably help resolve this issue, but such an approach is not scalable in most models. We expand on this discussion below, regarding gene replacement in *Drosophila*.
- Regarding whether ‘certain types of mutations’ cannot be accurately functionalized when studying supraphysiological PTEN. We agree with this statement. However, we would first comment that we took great strides to calibrate and benchmark the assay, and validated that supraphysiological PTEN did not cause significant problems interpreting the great majority of variants; otherwise we would have found substantial disagreement with other assays and the clinically classified variants. Second, every assay and every variant will have idiosyncrasies that make certain variants unreliable for functionalization in that specific assay. Moreover, even if a variant is found to be pathogenic on clinical genetic grounds, the aberrant mechanism of action is not always clear, and there can be idiosyncrasies in the mechanism of pathogenicity within the individual (or pedigree). This would cause discrepancies in the variant’s function in a well-established assay (outside the patient’s tissues). This seems to be the case for the R173H variant, discussed below. Indeed, in recognition that no assay nor clinical assertion could be 100% full proof in its assessment, Brnich et al (2019) included allowance for a degree of inaccuracy of a well-established assay in assessing pathogenic or benign variants. This is why we believe that multiple assays in multiple models should be established, in order to isolate situations where certain variants are incorrectly assessed by a specific assay. We have added considerable discussion of these points in the discussion starting at line 582.
- Overall, we believe that our assay makes a valuable contribution and fulfills the criteria for a well-established assay that has sufficiently high positive predictive value for pathogenic variants and negative predictive value for benign variants. We further believe that we make a substantive contribution as the first demonstration of this type of assay in *Drosophila*, a widely used model for human disease gene/variant analysis.

Reviewer 3: *“On page 13, the authors propose that the C124S mutant may interfere with normal PTEN function to boost PI3K signalling. This is an interesting and important idea, and could be tested by overexpressing this mutant, the wild type protein and a protein with no apparent activity (G129E) in the absence of activated PI3K. It would also be useful to test the novel putative interfering mutants, G127R and D22E, to confirm that they behave in the same ways, since identification of such mutants has been flagged as one of the advantages of the fly system. Has this putative dominant negative activity been suggested in any other system?”*

Reviewer 4: *A large number of the tested variants behave as dominant-negatives in the wing assay, likely impacted by this high level of expression. The variants would need to be expressed at endogenous levels, in a heterozygous or `homozygous dPTEN mutant background, to have informative predictive value. Minimally, testing and comparing effects at multiple expression levels is needed to convincingly demonstrate relevant function.”*

- Regarding dominant negativity, numerous variants indeed fall below the *attP2* (no PTEN) line, but most are not significantly different from *attP2*; we only found one variant that was significantly different from *attP2* controls, G127R (see line 410 in the manuscript and Fig. S4). While one mechanism for this may be interference with endogenous PI3K/AKT signaling via interaction with *Drosophila* Pten, this is unknown. The increase in pAkt levels when C124S was overexpressed is notable, and significant; however, C124S did not produce a significant difference in wing growth compared to *attP2*.
- While this issue is interesting, we do not consider this would be of general interest to most readers, nor an obstacle for our assay. These C124S and G127R variants were found to be a strong loss of function variants in all assays we compare our data to, so we successfully assessed this variant as pathogenic. Any inference of dominant negativity would not change this assessment.
- Pursuing studies of whether variants are truly act in a ‘dominant negative’ manner on fly Pten would require substantial physical and functional interaction studies between PTEN and Pten, in a context when both are expressed at the same levels. This is all beyond the scope of the current report. We also believe that this would be of only marginal interest to readers and would not help in determining whether any PTEN variants are antimorphic. We are considering allelic interaction studies to identify PTEN antimorphs in ongoing work in the fly model, but this is beyond the scope of the current manuscript. We have now discussed this starting at line 555.
- Regarding whether other assays identified variants as dominant negative. In our previous collaborative paper, we reported that a high number of variants were ‘dominant negative’ in a HEK cell assay, in the presence of endogenous PTEN. However, in this case, the overexpression of PTEN variants in that assay made it difficult to know if this reflected true antimorphic activity, as pointed out by reviewers here. The attempt to resolve this by eliminating endogenous PTEN, for a subset of variants, failed to test heteroallelic interactions require to identify antimorphs. We do not feel that the best venue for such analysis of a variant’s antimorphic activity is in any overexpression assay, which as the reviewers point out has too many confounds to make any reliable conclusions. Regardless, we do feel confident in saying that these variants exhibit a loss of function and are pathogenic, which is the primary goal for these assays.

“*For Fig. 2, it would have been informative to see how the effect of overexpressed human PTEN in the wing compared to Drosophila PTEN, so that it could be determined whether the human protein activity is similar to the activity of fly PTEN in Drosophila. Has this been done?”*

- We thank the reviewer for alerting us to this oversight. After reading all reviewer comments, we realized that these data are critical for the manuscript and are now included in Fig. S1 with discussion starting at line 312. In brief, we show that in fly tissues, PTEN is much less bioactive than fly Pten. This has implications for rescue experiments that reviewers suggested (discussed at length elsewhere here), and we apologize for this initial omission.

**Specific reviewer comments**

Reviewer #1

*“it raises the question of the true potential of Drosophila assays as an alternative.”*

- We would like to clarify that our goal is not to develop the ‘perfect’ assay that would serve as an alternative to other models, but to demonstrate the excellent performance of *Drosophila* models to functionalize human variants at scale, as a complement and extension to other models. We agree that we must be more careful to fully explain our goal in the manuscript. We believe it to be optimal to establish multiple well-established and well-validated assays for any gene in multiple models, and that *Drosophila* offers a particularly useful complementary assay platform. More extensive discussion of this point is provided starting at line 564.

*“Other comments are that the conclude that this assay can provide functional data for the interpretation of clinical variants for a large number of variants. However, the study provides a case for PTEN, but it lets open and there is not discussion about how generalizable and extensible is what they present to human proteins that are less either less conserved or have poor or completely unknown functions. That is the real challenge that the authors point out at the beginning of their introduction.”*

- This reflects a lack of clarity on our part, and we thank the reviewer for raising it. First, in such statements we are suggesting that *Drosophila* offers the capacity for assessing large numbers for variants for a single gene, if a well-established assay has been developed. Also, the major challenge we wished to address was the lack of evidence for the capacity of *Drosophila* to be used in scalable assays for clinical variant interpretation; as such we provide the first report of the successful functionalization of more than 12 variants (the previous highest number), in assays calibrated by over 11 total known pathogenic and benign variants. We have attempted to make this clearer in the introduction. Clearly, it is beyond the scope of this paper to establish assays for enough genes to determine which protein properties will best predict the successful use of *Drosophila* as a model, such as conservation, number of domains, type of enzymatic activity, etc. We have included a consideration of this topic in the revised discussion (lines 609).

*“An assay/test that is missing and easy is to test the PTEN variants to modify PI3KAct in the absence of endogenous Pten. This is relatively easy to do by starting the assays by constructing a stock with the gain of PI3K along with fly Pten RNAi or dCas9 / CRISPR and then express the PTEN variants. I was surprised that this test has not been implemented,”*

- We appreciate these suggestions. We did indeed consider a number of these approaches initially, but we felt that these approaches introduce as many confounds as they hope to resolve, and would seem unnecessary since our current assay as it accurately predicts the function of known variants and correlates extremely well with other assays.
- Knockdown by RNAi typically reduces, rather than eliminates the target protein, resulting in the confound of some unknown persistent level of Pten protein, that would differ in a fly culture-variable manner (temperature/humidity etc). This would reduce the reproducibility of the assay between labs and over time. Moreover, all available PTEN RNAi lines, even the Valium20 line, would be expected to potentially downregulate the transgenic PTEN transcripts, as all available RNAi lines target sequences that overlap with the human PTEN sequence. This would introduce a confound that would vary depending on each human variant’s unique sequence.
- The CRISPR approach would suffer from cell and homologous chromosome mosaicism, as well as culture condition variability between labs, in the ability to delete the endogenous Pten gene, and also introduces the confound of stochastic second site mutations. Validating the absence of Pten protein in every cell would be challenging and runs counter to our goal of scalability.

*“Another missing test/assay that the authors could have explored is a knock-in in which the fly Pten is replaced by the PTEN, so that researchers can assay function in a more physiological context without the overexpression resulting from the Gal4/UAS.*

*A knock in seems a strategy that may be more predictive and less prone to overinterpretation of, for example, gain of function. Clearly this is more time consuming but it could be discussed in the Discussion section as a future tool/approach to avoid supranormal level of human proteins which may in some cases be toxic to Drosophila —personal experience.”*

- We appreciate this comment, and the reviewer’s acknowledgement that this is more of a discussion point than a request. We would comment that it is was not our goal to develop an assay for how well PTEN functions in *Drosophila* tissue, or how PTEN variants rescue fly Pten activity; rather, we wished to set up an assay that explicitly shows how PTEN suppresses PI3K/AKT signaling in tissue growth. Moreover, as the reviewer appreciates, an assay that would require placing every *UAS-PTEN* variant transgene into a *Pten* null allele would greatly compromise the scalability of the assay, and scalability is a primary goal. This has been addressed in line 266.
- We find that PTEN has significantly less bioactivity that Pten in the fly. Expression of fly Pten in the wing using *omb-Gal4* caused a more dramatic reduction in wing size compared to PTEN and this is now included in the results section starting line 312 and shown in Fig. S1A, B. Similarly, fly Pten greatly suppressed the enlarged wing size in comparison to PTEN, in an activated PI3K background, as shown in Fig. S1C, D. However, importantly, we previously showed that PTEN in the fly is not toxic even when expressed ubiquitously (Post et al, 2020). Here we show it can rescue an overgrowth phenotype in *Pten* hypomorphs and that it can suppress a gain of function PI3K phenotype back to the near wildtype wing size. None of these factors indicates that PTEN supraphysiological levels is a major confound in our assay.
- *Pten* nulls are embryonic lethal. This is reported to be due to impacts on PI3K signaling but also Pten-dependent organization of the actin cytoskeleton (now in results line 254). Thus, any rescue by PTEN would not explicitly test the defined gene-disease mechanism of PI3K/AKT pathway suppression. Further, the relative low bioactivity of PTEN may result in a failure to rescue viability (see line 314). Yet, even if PTEN could rescue viability in such a scenario, loss of function variants would presumably cause lethality. Such a binary lethal/survival assay output would fail to discriminate relative function of many variants, which our reported assay does very well. One option would be to employ a labor-intensive timing of lethality, but this compromises scalability and ease of implementation. It would also bin data into 12 or 24 hour brackets, resulting in a loss of the continuous quantitative data we obtain for the wing assay. It would also not be able to discriminate the relative function of normally functioning variants. Finally, we found that gain of function variants like 4A were also lethal; in which case a lethality-based assay would inappropriately define it as loss of function.

*“My impression was that the goal has been to establish Drosophila as "the" system scalable to test batches of human variants together in the same setting but it seems that the goal is to use Drosophila assays as an additional toolkit. Perhaps clarifying these ideas of the discussion in the introduction would work better.”*

- We appreciate the reviewer’s thoughtful comments. We have endeavored to clarify that we do not propose a fly assay as the only one that is worth performing, but instead that it offers a reliable tool for large scale variant assessment to complement and extend other well-established assays in support of a function call for any single variant. These statements are throughout the manuscripts

*“I also missed a more critical evaluation of the advantages but also the limitations of the Drosophila paradigm based on the data obtained but also thinking on less conserved or conserved but less well functionally characterized human proteins that may be relevant for clinical interpretations and of interest for the non-specialists to evaluate the usefulness of Drosophila.”*

- We agree and thank the reviewer for pointing this out. We hope the section starting line 609 resolves this concern.

**Reviewer #2:** *“Based on the Methods section, it seems that the transgenes used to test 97 variants (93 from patients and 4 biochemical) were reported in their previous study (Post et al., 2020 Nature Comm). However, I don’t see a direct comparison of the wing assay used in this paper with their previous functional assay (lethality/growth based on overexpression in otherwise wild-type animals). Were all the results consistent, or were there any outliers between the two fly assays? Since they do a comparison between their fly wing assay, yeast assay, human cell- based assay and in silico predictions, I feel it is worth discussing what variants were missed in the previous growth-based assay that is not picked up in the wing assay and vice versa.”*

- This is an excellent suggestion that we overlooked. We now address this starting at line 440 and also Figs. S6 and S7.

*“I feel the rational of why the authors feel there is a need to develop a fly-based assay are explained in diverse places within the paper, but I feel it would be nice to consolidate/re-emphasize it in one or two paragraphs in a logical fashion. I feel the section in the discussion that starts with Line 387 “No single assay can capture all aspects of the entire functionality of all PTEN variants, even in human cell or organoid models…” and Line 415 “First, each assay or model has relative strengths and weaknesses, and each assay will assess distinct…” has very important messages and explains that the assays that the author developed is complimentary to the other functional assays. By emphasizing these points more in their text, I feel that many more readers will recognize the significance of their research.”*

- We thank the reviewer for this excellent suggestion. This was indeed lacking and makes an important point that will strengthen the manuscript and address concerns of other reviewers well. We hope we have addressed this sufficiently throughout the discussion.

**Reviewer #3:**

“*For Fig. 2, it would have been informative to see how the effect of overexpressed human PTEN in the wing compared to Drosophila PTEN, so that it could be determined whether the human protein activity is similar to the activity of fly PTEN in Drosophila. Has this been done?”*

- We thank the reviewer for alerting us to this oversight. After reading all reviewer comments, we realized that these data are critical for the manuscript and are now included in Supplementary Fig. 1 (Fig. S1). In brief, we show that in fly tissues PTEN is less bioactive than fly Pten.

*“Page 9 – in the comment on Fig. 2A, B (line 160), it seems that overexpressed PTEN actually reduces growth below wild type levels, rather than just fully suppresses the activated PI3K phenotype. This should be mentioned because it suggests that the 100% activity value involves a subtle reduction in wing growth when combined with activated PI3K, a weaker version of the 4A mutant’s effect.”*

- We agree with the reviewer and have added this observation to the manuscript (line 282).

**Reviewer #4:**

*“The assays described here measure the relative ability of PTEN variants to suppress the effects of overexpressed, constitutively activated PI3K, which leads to an enlarged wing when driven by the omb-GAL4 driver. This would seem to be an appropriate context for PTEN to function in suppressing oncogene-driven tumorigenesis, but likely does not reflect its action in cells with normal growth factor signaling. A focus on the ability of human PTEN variants to rescue Drosophila PTEN mutant phenotypes, such as pupal overgrowth, would be a more physiologically relevant test of variant function.”*

- The reviewer alerts us to an important point that we inadequately addressed in the manuscript. We have addressed the issue of ‘physiological context’ in our response to reviewer 1 and added text in the discussion to address this point. We respond to the specific comment as follows: first, the rescue of pupal overgrowth assay is not scalable due to the need to place the *UAS-PTEN* transgenes in a *Pten* allele background. Further, the ClinGen SVI guidelines recommend gene-disease-relevant context, and not how well a human gene performs the function of an orthologous gene in a model system context. As such, we explicitly tested PTEN’s ability to suppress PI3K/AKT pathway tissue overgrowth, rather than pupal overgrowth, which does not discretely test the defined gene-disease function of PTEN, and as such would not align with SVI guidelines.
- We have now added new data to the paper showing that PTEN is considerably less bioactive than *Drosophila* Pten. Thus, because *Pten* nulls are embryonic lethal (note that the rescue experiments we performed were in hypomorphs with some level of endogenous Pten), a whole organism rescue assay would most likely result in either lethality or other considerable complexities that would preclude our ability to test variants in a continuous quantitative manner. Even if we were to introduce CRIMIC-type swappable interchangeable cassettes (SICs) into the *Pten* locus, every integrant variant would have to be sequence verified and made homozygous before testing. This is a major impediment to making this assay scalable given the labor involved. Moreover, even if we were to assume that a benign or wildtype PTEN integrant resulted in viability to adulthood, the loss of function (pathogenic) variants would not, resulting in lethality that would not allow us to accurately assess relative variant function in a continuously quantitative phenotype. In this case, we would have to quantify time to lethality (with whichever non-continuous timepoints we chose), which again would not explicitly test disease-relevant suppression of PI3K/Akt pathway activity and would be very laborious.

*“The authors show that selected groups of 14 pathogenic and 11 nominally functional variants segregate into discrete groups in the wing overgrowth assay, and they use these groups to set scoring thresholds to sort a collection of PTEN variants. In scoring these remaining test variants, however, they fail to assess whether these scores actually provide useful information – whether known pathogenic variants fall below the cutoff and benign variants above it. A cursory examination of several alleles suggests that this is not the case. For example, the R173H variant is categorized as pathogenic by ClinVar based on multiple lines of evidence, but it falls well above the benign cutoff as shown in Figure 8. Another pathogenic variant, T202I, lies just at the benign threshold. These examples would seem to call into question the confidence one could place in results based on this assay, and they highlight the need for further improvement of this model.”*

- We have now provided a text in the results section (line 496) and a graph, Fig. S8, showing that our assay performs well for ClinVar pathogenic variants, and against other assays for the same variants. For PTEN expert panel curated variants, which represents the highest level of confidence that the variant is pathogenic, we find that our assay finds all to be <46% of wildtype activity. This matches well with the PTEN expert panel recommendation that variants exhibiting <50% activity be considered as pathogenic. For variants supported by ‘multiple submitters’, we show that all variants except R173H are loss of function in our assay. R173H is an intriguing variant that is mostly found to have wildtype activity in most assays. Therefore, we believe that this variant exhibits certain idiosyncrasies that make it challenging to functionalize, in any assay. This has now been included in results section (line 507) and reiterated in the discussion (line 586). The other pathogenic variants are submitted by ‘single submitters’, which are known to be less reliable. Moreover, all assays showed a range of abnormal to normal assessments for these variants that were mostly consistent across assays. The T202I variant is a single submitter variant and all other available assays show it to be benign, in agreement with our assay. This is highlighted in line 517.
- Overall, we completely agree that we must be more careful to fully explain these factors in the manuscript. However, uniquely in the field of PTEN variant functionalization to date, we have endeavored to calibrate our experimental assay to the highest standards of practice and find that our assay performs very well. Therefore, as much as one can believe that any calibrated assay provides useful information, this assay performs well. Finally, we would add that our goal is not to develop the ‘perfect’ assay but demonstrate the excellent performance of *Drosophila* models to functionalize human variants at scale.

*“Have all data underlying the figures and results presented in the manuscript been provided?*

*Large-scale datasets should be made available via a public repository as described in the PLOS Genetics data availability policy, and numerical data that underlies graphs or summary statistics should be provided in spreadsheet form as supporting information.”*

- We included all raw and normalized datasets into Supplemental Table 3 (Table S3).

*“The authors fail to demonstrate predictive power of their assay on an untrained data set, which further limits the applicability of their findings.”*

- Overall, in order to achieve our primary goal of demonstrating the reliability of a *Drosophila* model for variant functionalization and clinical interpretation, we had to by necessity use trained datasets as per SVI guidelines. However, our experimenters were blinded to the variant’s identity when datasets were generated and distributed; we only found out their relative activity afterwards, as outlined in methods. Then, only after correlation analysis, did we find that our data correlated very well against a HEK dataset which our collaborative paper previously performed. It will be fascinating in the future to determine whether variants we interpret as pathogenic prove to be pathogenic on the basis of future clinical evidence. Alternatively, obtaining an untrained dataset would require clinicians (or preferably the PTEN expert panel) to withhold evidence of pathogenicity for many more variants, until after we performed our assay. This would be beyond the scope of our current report. Overall, we report a successfully calibrated assay that performs very well against known variants and other assays, using the guidelines recommended for such assays.

Additional Notes.

- As we reviewed all our data for the revision, we noted an error in unblinding of two variants, R130L and D252G. All stocks were assigned numbers that did not reveal their variant identity, to assure blinding in scoring crosses. R130L was labelled as 644 and D252G was labelled as 649. At some point these labels got switched because of the resemblance in the way that 4 and 9 were written by hand. We re-sequenced these *Drosophila* strains, and confirmed this error. We have adjusted our datasets and graphs accordingly.

Yours sincerely

Douglas Allan, PhD

Associate Professor

Department of Cellular and Physiological Sciences

Room 2401 Life Sciences Centre

2350 Health Sciences Mall

University of British Columbia

Vancouver, British Columbia

CANADA (V6T 1Z3)

Office Phone: (604) 827-5960

Lab Phone: (604) 827-4159

Fax: (604) 822-2316
